# Nutritional and antioxidant changes in lentils and quinoa through fungal solid-state fermentation with *Pleurotus ostreatus*

**DOI:** 10.1186/s40643-022-00542-2

**Published:** 2022-05-11

**Authors:** J. Sánchez-García, A. Asensio-Grau, J. García-Hernández, A. Heredia, A. Andrés

**Affiliations:** 1 Instituto Universitario de Ingeniería de Alimentos Para el Desarrollo (IIAD), Universitat Politècnica de València, Camino de Vera s/n, 46022 Valencia, Spain; 2 Centro Avanzado de Microbiología de Alimentos (CAMA), Universitat Politècnica de València, Camino de Vera s/n, 46022 Valencia, Spain

**Keywords:** Seeds, Flour, Protein, Polyphenols, Antinutrients

## Abstract

Solid-state fermentation (SSF) may be a suitable bioprocess to produce protein-vegetal ingredients with increased nutritional and functional value. This study assessed changes in phenol content, antinutrient content, biomass production and protein production resulting from the metabolic activity of *Pleurotus ostreatus*, an edible fungus, in lentils and quinoa over 14 days of SSF. The impact of particle size on these parameters was also assessed because the process was conducted in both seeds and flours. Fungus biomass increased during fermentation, reaching 30.0 ± 1.4 mg/g dry basis and 32 ± 3 mg/g dry basis in lentil grain and flour and 52.01 ± 1.08 mg/g dry basis and 45 ± 2 mg/g dry basis in quinoa seeds and flour after 14 days of SSF. Total protein content also increased by 20% to 25% during fermentation, in all cases except lentil flour. However, the soluble protein fraction remained constant. Regarding phytic acid, SSF had a positive impact, with a progressive decrease being higher in flours than in seeds. Regarding antioxidant properties, autoclaving of the substrates promoted the release of polyphenols, together with antioxidant activity (ABTS, DPPH and FRAP), in all substrates. However, these parameters drastically decreased as fermentation progressed. These results provide scientific knowledge for producing lentil- or quinoa-based ingredients with low antinutrient content enriched with protein fungal biomass.

**Graphical Abstract:**

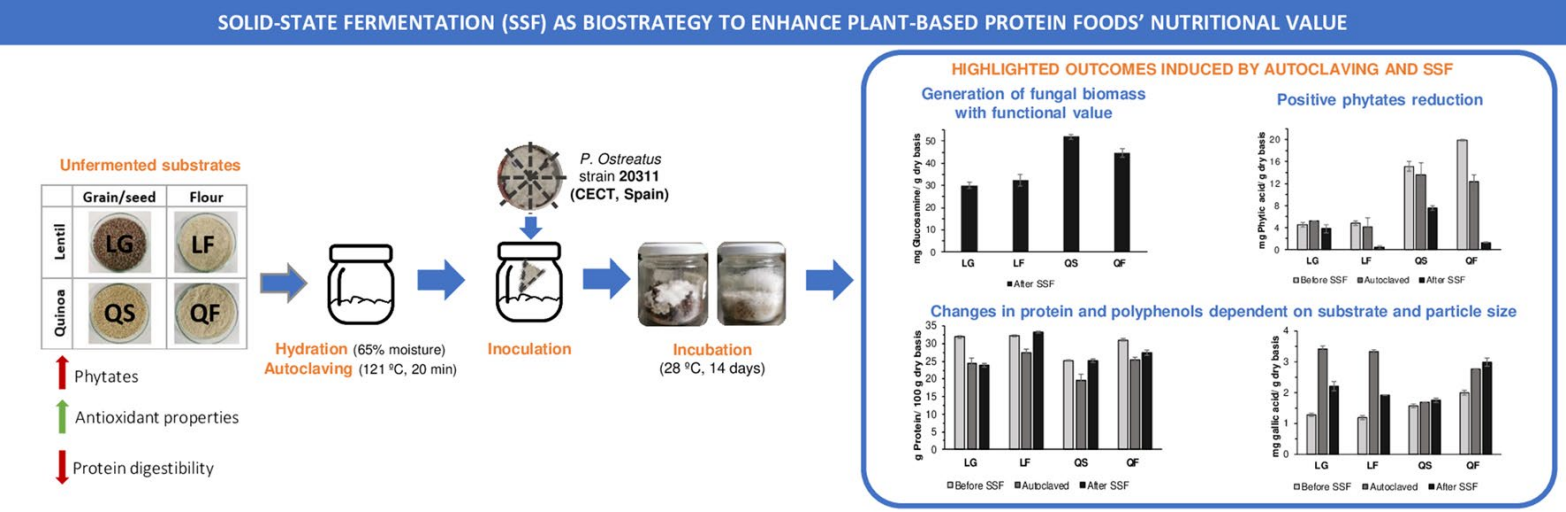

## Introduction

The growing interest in seeking plant protein sources as an alternative to animal proteins is driven by environmental sustainability, cost and food security motivations. Both legumes and pseudocereals are relevant for agriculture and food security because of environmental and economic benefits associated with their ability to fix atmospheric nitrogen in soils (Khazaei et al. 2019). This fact contributes to mitigating greenhouse gas emissions and thus reducing the need for external nitrogen fertilisers (Nemecek et al. 2008; Sánchez-Navarro et al. 2020).

Legumes are one of the most consumed foods worldwide. They are an essential component not only of the Mediterranean diet but also of the diet in many developing countries (Clemente and Jimenez-Lopez 2020). Lentils, chickpeas, beans and peas, amongst other legumes, are rich sources of protein and complex carbohydrates such as insoluble fibre, which has a low glycaemic index (Bouchenak and Lamri-Senhadji 2013; Dhull et al. 2020). They also have a high content of bioactive compounds, such as B vitamins, minerals such as potassium and magnesium, and polyphenols (Becerra-Tomás et al. 2019; Khazaei et al. 2019). Lentils (*Lens culinaris*) in particular are frequently noted for their protein content, essential micronutrients and antioxidants (Khazaei et al. 2019). The presence of phenolic compounds and precursor proteins of bioactive peptides, known as bioactive molecules, provide them with antioxidant and antidiabetic activities of considerable interest (Magro et al. 2019). Another interesting food group is that of pseudocereals, such as amaranth, buckwheat and quinoa, which differ from cereals in some morphological properties and their distinct chemical composition (e.g. they are high protein and gluten free). Specifically, quinoa (*Chenopodium quinoa Willd*), a crop from the Andean region, is one of the grains of the twenty-first century. Its cultivation has now spread to European countries, the United States and Canada (Romano and Ferranti 2019). It is a source of high-quality protein that contains the nine essential amino acids, with a high content of lysine, methionine and cysteine in comparison with common cereals (Motta et al. 2019). Despite the high-quality nutritional profile of lentils and quinoa, they also contain antinutrients (phytates, polyphenols, such as tannins, and gastric protease inhibitors), which hinder digestibility and the absorption of nutrients (Schlemmer et al. 2009; Nkhata et al. 2018; Asensio-Grau et al. 2020). Phytates mainly affect the bioavailability of minerals, as may also occur with tannins (Bouchenak and Lamri-Senhadji 2013; Khazaei et al. 2019). Tannins react with amino acids, such as lysine and methionine, limiting their bioavailability (Sarwar-Gilani et al. 2012; Samtiya et al. 2020). Protease inhibitors irreversibly alter gastric proteases, such as trypsin, leading to a decrease in protein digestion and amino acid absorption (Khazaei et al. 2019).

Cooking methods are known to reduce the negative impact of antinutrients and improve food digestibility (Muzquiz et al. 2012; Shi et al. 2017). Many of these molecules, such as protease inhibitors, are thermosensitive, whereas others, such as tannins, saponins and phytates, can be reduced by soaking, germination or even fermentation (Muzquiz et al. 2012). Fermentation is a biological process that entails the conversion of substrates into new added-value products through the metabolic actions of microorganisms. Compared with their non-fermented counterparts, the resulting fermented foods have improved nutritional composition and functionality thanks to the hydrolysis of complex macromolecules (fats, carbohydrates and proteins) into low molecular weight compounds that are likely to be easier to digest and can be further bioabsorbed (Gupta et al. 2018; Şanlier et al. 2019). This improved digestibility is especially relevant for some population groups suffering from gastrointestinal disorders, such as pancreatic insufficiency and for vegans, whose main protein intake comes from vegetables.

Solid-state fermentation (SSF) offers an environmentally and economically sustainable alternative to classical liquid-state fermentation (submerged method, SmF). SSF occurs in the absence of free water, and the microorganism is in direct contact with gaseous oxygen (Raghavarao et al. 2003). In addition, SSF allows fermentation in a wide variety of substrates that may also be very cheap, such as agro-industrial waste. Furthermore, SSF reaches higher final product concentrations since enzymes inhibition is scarce; SSF would convert 20–30% of the substrate, whereas in SmF the maximum amount is around 5% (Liu and Kokare 2017). The potential benefits of SSF have been described in relation to the revaluation of industrial by-products, such as the production of ethanol from lignocellulosic waste (Raghavarao et al. 2003; Gupta et al. 2018). SSF also represents a major advance in the production of protein-enriched foods from carbohydrate-rich substrates (Raghavarao et al. 2003). Moreover, scholars have reported the positive effect of SSF on the nutritional profile of legumes, such as chickpeas (Xiao et al. 2014), beans (Espinosa-Páez et al. 2017) and lentils in both grain (Dhull et al. 2020) and flour (Magro et al. 2019). Temperature, humidity, available gases and pH, together with inoculum selection, are some of the key processing variables to optimise SSF processes (Pandey 2003).

The employment of different microorganisms has been reported in the SSF of dietary substrates (Couto and Sanromán 2006). In particular, mushrooms are considered a high nutritional value source due to their content of carbohydrates, essential amino acids, fibre, vitamins and minerals (Espinosa-Páez et al. 2017). Their potential medicinal and pharmacological benefits are also well documented (Atlý et al. 2019). The genera *Ganoderma, Lentinula, Trametes, Cordyceps, Hericium* and *Pleurotus* are notable examples (Atlý et al. 2019). The edible species of the genus *Pleurotus*, catalogued as Generally Recognized As Safe (GRAS), have been highlighted by several authors for their ability to synthesise essential amino acids whilst developing characteristic organoleptic properties (Espinosa-Páez et al. 2017). The *Pleurotus ostreatus* species is one of the most commonly grown and produced species worldwide. This mushroom is capable of growing on lignocellulosic substrates, which makes it especially suitable for the degradation of substrates, such as legumes, seeds and grains.

The aim of this study was to analyse the impact of solid-state fermentation (SSF) with *P. ostreatus* on protein, phytate and polyphenol contents, as well as antioxidant activity, in lentil and quinoa substrates.

## Materials and methods

### Materials

Lentil (*Lens culinaris*) of the “pardina” variety and quinoa (*Chenopodium quinoa Willd.*) grains/seeds and flours were acquired from Molendum ingredients S.L. (Batch: 19011573). The *Pleurotus ostreatus* strain was obtained from the Spanish Type Culture Collection (CECT; 20311; batch: 18-10-2016) from the Universitat de València (Valencia, Spain). To formulate the culture media, malt extract, glucose, mycopeptone and agar powder were supplied by Scharlab (Barcelona, Spain).

The analytical determinations required the following reagents: sodium hydroxide (NaOH), acetylacetone (Ce_5_H_8_0_2_), ethanol (CH_3_OH), methanol (CH_3_CH_2_OH), gallic acid (C_7_H_6_O_5_), Trolox (C_14_H_18_O_4_), DPPH reagent (C_33_H_44_N_5_O_6_), iron chloride hexahydrate (FeCl_3_ (III)·6H_2_O), TPTZ reagent (C_18_H_12_N_6_), acetic acid (C_2_H_4_O_2_), ABTS reagent (C_18_N_24_N_6_S_4_), Folin-Ciocalteu reagent, thioglycolic acid (C_2_H_4_O_2_S), potassium persulfate (K_2_S_2_O_8_), calcium chloride dihydrate (CaCl_2_·2H_2_O), *p*-dimethylamine benzaldehyde (C_9_H_11_NO) and glucose (C_6_H_12_O_6_). These reagents were acquired from Sigma-Aldrich (St. Louis, MO, USA). The total starch kit (AA/AMG) was obtained from Megazyme (Ireland). Glucosamine (TCI Chemicals, USA), acetylacetone (C_5_H_8_O_2_), sulphuric acid (H_2_SO_4_), ammonium iron sulphate (NH_4_Fe (SO_4_)_2_·12H_2_O) and hydrochloric acid (HCl) were acquired from AppliChem Panreac (USA). Sodium phytate was acquired from Biosynth Carbosynth (USA). Sodium carbonate (Na_2_CO_3_) was acquired from Scharlab (Barcelona, Spain).

### Fungal solid-state fermentation (SSF)

#### Starter culture preparation

*Pleurotus ostreatus* colonies were isolated from the agar plate and cultured in agar petri dishes made with 2% glucose, 2% malt extract, 0.1% mycopeptone and 1.5% agar. They were then incubated for 14 days at 28 °C (Selecta J.P. 200207, Germany). The resulting mycelium was inoculated with a loop in the culture broth (2% glucose, 2% malt extract and 0.1% mycopeptone) and incubated again at 28 °C for 14 days. This broth was used as the starter culture for fermentation. For the preparation of the starter culture, glass petri dishes containing 10 g of lentil or quinoa flour with 65% of moisture were sterilised (121 °C, 20 min), inoculated with 1 ml of *Pleurotus ostreatus* in the previously prepared liquid medium and incubated at 28 °C for 14 days until the lentil or quinoa surface was completely colonised by the mycelium.

#### Fermentation process

Lentil (grain and flour) and quinoa (seeds and flour) were subjected to fungal SSF as described by Asensio-Grau et al. (2020), with some modifications. Glass jars (250 ml) containing 35 g of grain or flour were moistened to 65% (for grain and flour, a distilled water proportion of 1:0.65 (w/v) was used) and sterilised at 121 °C for 20 min. Then, the glass jars were inoculated with one portion of the starter culture previously divided into eight portions. Finally, the glass jars were incubated at 28 °C for 14 days. Three glass jars were taken at each of the fermentation times 0, 2, 4, 6, 8, 10, 12 and 14 days to conduct the corresponding analytical determinations.

### Analytical determinations

#### Substrate composition

Protein, lipid, ash and moisture contents were determined by the AOAC methodologies in lentil and quinoa (AOAC 2000). Carbohydrates were estimated by subtracting lipid, protein and ash contents from the total solid content.

#### Fungus biomass

Glucosamine content was used to estimate fungus growth, considering glucosamine, such as a product of the chitin hydrolysis (Aidoo et al. 1981; Tomaselli Scotti et al. 2001). For fungal chitin hydrolysis into *N*-glucosamine, 100 mg of dried lentil and quinoa samples was incubated with 2.4 ml of 72% sulphuric acid (H_2_SO_4_) at 25 °C for 24 h. Then, samples were diluted with 55 ml of distilled water. The hydrolysis was carried out by sterilising the sample for 2 h at 121 °C. The hydrolysed products were neutralised to pH 7 using sodium hydroxide (NaOH) 10 M and 0.5 M. Next, 1 ml of hydrolysed product was added with 1 ml of acetylacetone reagent (1 ml of acetylacetone and 50 ml of sodium carbonate 0.5 M) in glass tubes and incubated in a boiling water bath for 20 min. After cooling the tubes, 6 ml of ethanol and 1 ml of Erhlick reagent (2.67 g *p*-dimethylamine benzaldehyde and ethanol:HCl solution 1:1 (v:v)) mixed into a 100-ml volumetric flask were added to the mixture. Then, the samples were incubated at 65 °C for 10 min, and absorbances were measured at 530 nm using a spectrophotometer (Thermo scientific, Helios Zeta UV/Vis). A calibration line taking glucosamine (0–0.5 mg/ml) as standard was used to quantify the fungus biomass. Results are expressed as milligrams of glucosamine per gram of dry basis.

#### Protein content

Protein content was determined by the Kjeldahl method following AOAC methodologies (AOAC 2000). Results are expressed as grams of protein per 100 g of dry basis.

#### Trichloroacetic acid (TCA) soluble protein

Amino acids released during fermentation were estimated as the amount of soluble protein in trichloroacetic acid (TCA) following the method described by Asensio-Grau et al. (2020) and Gallego et al. (2021). Samples (100 mg) were mixed with TCA solution to a final concentration of 12% and incubated at 4 °C for 15 min. Then, samples were centrifuged (Eppendorf MiniSpin Plus) at 4200*g*-force for 10 min. The supernatant was diluted with 50 mM EDTA and 8 M UREA buffer (pH 10), and the absorbance was measured by ultraviolet spectrophotometry (Helios Zeta UV/Vis, Thermo Scientific) at 280 nm. A calibration line was used for quantification using tyrosine as standard. Results are expressed as grams of soluble protein in TCA per 100 g of protein.

#### Phytate content

Phytate content was determined using the method published by Haug and Lantzsch (1983) and adapted from Peng et al. (2010). This method is based on the precipitation of phytic acid using an acidic iron solution. The decrease of iron in the supernatant is proportional to the amount of phytic acid in the sample. Ferric solution (0.2 g of NH_4_Fe (SO_4_)_2_·12H_2_O in 100 ml HCl 2 M, with the volume raised to 1000 ml with distilled water) and bipyridine solution (1 g 2,2-bipyridine and 1 ml of thioglycolic acid, with the volume raised to 100 ml with distilled water) were prepared in advance. For the analysis, 50 mg of the sample was extracted with 10 ml HCl 2 M overnight at 4 °C. Then, the samples were vortexed, and 0.5 ml of the extract was added to a capped glass tube with 1 ml of ferric solution. The samples were then placed in a boiling water bath for 30 min. After cooling the samples to 25 °C, 2 ml of bipyridine solution was added, and the samples were vortexed and immediately measured by spectrophotometry at 519 nm (Helios Zeta UV/Vis, Thermo Scientific). For quantification, a calibration line was produced using phytic acid as standard (0–0.15 mg/ml). Results are expressed as milligrams of phytic acid per gram of dry sample.

#### Total polyphenols

Polyphenols were determined in samples using the Folin–Ciocalteu method following the indications of Espinosa-Páez et al. (2017) and Chang et al. (2006). An extraction with 80% methanol for 2 h in agitation (55 rpm, 25 °C, Intelli-Mixer RM-2) was performed to recover the hydrosoluble compounds from the samples. Methanol was added to the sample in a proportion of 1:20 (w:v). After agitation, samples were centrifuged (20 min, 14*g*-force, 20 °C), and the supernatant was used to quantify the polyphenols by visible spectrophotometry (Helios Zeta UV/Vis, Thermo Scientific). A gallic acid line was used to quantify the total polyphenols (0–200 mg/l). Results are expressed as milligrams of gallic acid per gram of dry basis.

#### Antioxidant activity

Three methods were used to measure antioxidant activity in fermented samples following the indications of Thaipong et al. (2006) and Espinosa-Páez et al. (2017): (1) ABTS: 2,2′-azino-bis (3-ethylbenzothiazoline-6-sulphonic acid); (2) DPPH: 2,2-diphenyl-1-picrylhydrazyl and (3) FRAP: Ferric reducing antioxidant power. An extraction with 80% methanol was conducted to determine antioxidant activity. After centrifugation, supernatants were used for quantification using a spectrophotometer (Helios Zeta UV/Vis, Thermo Scientific). In all methods, a calibration line was required using Trolox as standard (0–200 mg/l). Results are expressed as milligrams of Trolox per gram of dry basis.

### Statistical analysis

Simple factor analysis of variance (ANOVA) was performed with a confidence interval of 95% (*p* < 0.05) to study possible differences in structure (between grain/seeds and flour) and fermentation time (days). The statistical program Statgraphics Centurion-XV was used for this purpose. Fermentation and analyses were performed by triplicate.

## Results and discussion

Lentils and quinoa can be considered good providers of nutrients for microorganism growth in fermentative processes. However, any modification (chemical or physical) of the starting substrate could affect the fermentative process, even when the same microbial species is used (Michael et al. 2011; Limón et al. 2015; Espinosa-Páez et al. 2017). Table 1 shows the nutritional composition (in dry basis) of lentils and quinoa, before (grain/seed) and after milling and sieving (flour). Quinoa is richer in lipids, minerals, phytates and phenols than lentils. Regarding protein content, all substrates had more than 30 g per 100 g of dry basis, except quinoa grain, which had a lower protein content. The removal of some fibrous parts of the quinoa seeds during milling and after sieving may be responsible for the differences between seeds and flour in terms of protein content, as well as carbohydrate and lipid contents. The antioxidant activity values (mg Trolox/g dry basis) of the substrates based on radical-based scavenging assays 2,2′-azino-bis (3-ethylbenzothiazoline-6-sulphonic acid (ABTS) and 2,2-diphenyl-1-picrylhydrazyl (DPPH)) and a non-radical redox potential-based scavenging assay (FRAP) also appear in Table 1. According to the results, higher values were observed in the ABTS assay than in the DPPH and FRAP assays, regardless of the substrate. Moreover, lentils had a slightly higher capacity to quench the ABTS and DPPH radicals than quinoa, despite the lower phenolic content of lentils. A positive relationship between total phenolic content and radical-based scavenging assays has been reported in vegetal foods (Marathe et al. 2011; Devi et al. 2019). In this study, this relationship seems to be related to the phenolic profile rather than the total content. Accordingly, phenolic compounds from lentils exhibited higher antioxidant activity than those from quinoa. Chemical species with hydrogen atom or electron donating ability exert antioxidant properties. In the case of phenols, these capabilities seem to be related to the position and number of hydroxyl groups attached to the aromatic rings. Catechin and proanthocyanidin compounds represent 69% of the identified phenols in pardina lentils (Aguilera et al. 2010). Phenolic acids together with flavanols comprise 60% of total compounds in white quinoa (Rocchetti et al. 2019). No statistically significant differences were found amongst samples regarding the capacity to reduce the ferric 2,4,6-tripyridyl-s-triazine complex [Fe^3+^−(TPTZ)_2_]^3+^ from ferric iron (Fe^3+^) to ferrous iron (Fe^2+^) in acidic medium (Table 3).

**Table 1:** Composition and antioxidant activity of lentil grain, quinoa seeds and respective flours

	Lentil grain (LG)	Lentil flour (LF)	Quinoa seeds (QS)	Quinoa flour (QF)
Protein	31.9 ± 0.5^A^	32.3 ± 0.3^A^	25.2 ± 0.2^A^	31.1 ± 0.3^B^
Lipids	0.86 ± 0.08^A^	1.19 ± 0.10^B^	3.4 ± 0.3^A^	8.6 ± 0.3^B^
Ash	2.76 ± 0.09^B^	2.58 ± 0.05^A^	3.60 ± 0.01^A^	4.09 ± 0.02^B^
Carbohydrates	64.5 ± 0.7^B^	63.9 ± 0.4^A^	67.7 ± 0.6^B^	56.2 ± 0.7^A^
Moisture	10.15 ± 0.05^B^	9.35 ± 0.02^A^	10.34 ± 0.09^B^	8.97 ± 0.05^A^
Phytic acid content	4.5 ± 0.4^A^	4.8 ± 0.4^A^	15.2 ± 0.9^A^	19.9 ± 0.2^B^
Total phenolic content	1.28 ± 0.05^A^	1.19 ± 0.07^A^	1.57 ± 0.06^A^	2.00 ± 0.08^B^
Antioxidant activity (ABTS)	3.8 ± 0.2^A^	3.5 ± 0.2^A^	2.4 ± 0.2^A^	3.1 ± 0.2^B^
Antioxidant activity (DPPH)	1.26 ± 0.09^A^	1.10 ± 0.06^A^	0.82 ± 0.05^A^	0.94 ± 0.04^B^
Antioxidant activity (FRAP)	2.09 ± 0.10^A^	1.8 ± 0.2^A^	1.8 ± 0.2^A^	2.3 ± 0.2^B^

Results are expressed in g/100 g dry basis for proximate composition, mg phytic acid, gallic acid or Trolox/g dry basis, phytic acid content, total phenolic content and antioxidant activity, respectively. They represent the mean of three repetitions with their standard deviation. ^AB^ Capital letters indicate significant differences between grain/seeds and flour at the 95% (*p* < 0.05) significance level

**Fig. 1:**
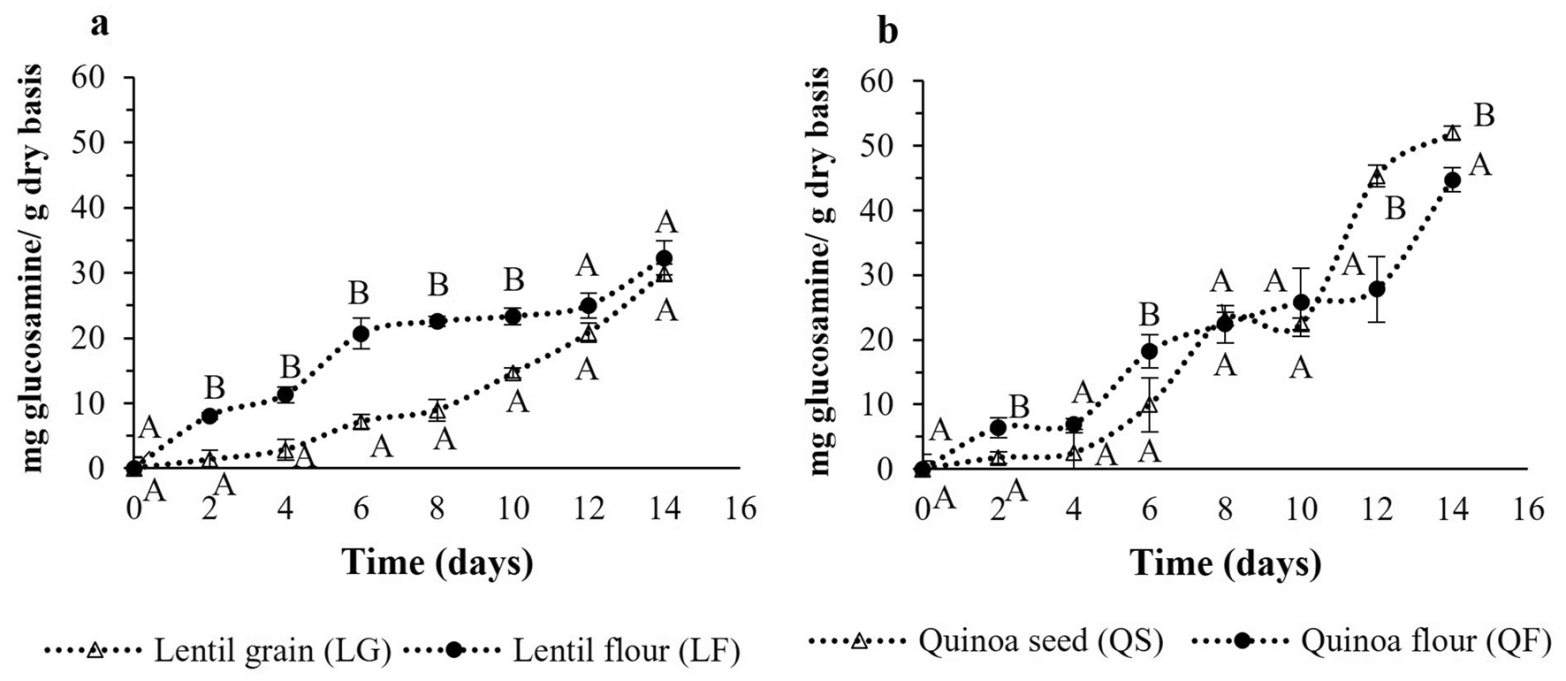
Biomass production in lentil grain, quinoa seeds and respective flours at different fermentation times. ^AB^ Capital letters indicate significant differences between grain/seeds and flour at the 95% (*p* < 0.05) significance level

The evolution of the fermentation process was followed by estimation of the unicellular biomass generation in the medium. Fungal biomass is difficult to assess because fungal cells do not easily separate from the solid substrate. The measurement of glucosamine (chitin monomer and a major constituent of the cell wall in fungi) is an acceptable indicator for the estimation of fungal mycelium development (Tomaselli Scotti et al. 2001). The biotransformation of the substrate using *P. ostreatus* depends on its ability to grow and secrete certain enzymes (mainly oxidative and hydrolytic) able to metabolise substrates rich in lignocelluloses (Rodrigues Da Luz et al. 2012), which are not directly fermentable. White rot fungi, such as *P. ostreatus,* have two types of extracellular enzyme systems: a hydrolytic system that produces hydrolases responsible for the degradation of polysaccharides and an extracellular and oxidative lignolytic system that degrades lignin (Ergun and Urek 2017). The growth of *P. ostreatus* (CECT 20311) observed by monitoring the evolution of glucosamine content is affected not only by the amount of nutrients but also by the morphological characteristics (i.e. grain or flour) of the substrate (Fig. 1). The initial section of the curve, between day 0 and day 4, corresponds to the latency phase of the fungus. An exponential increase in the growth of the mycelium began on the 4th day of fermentation, without reaching a stationary phase during the observed period. Despite some observed differences in the growth rate between grains and flours, similar values were found after 14 days of incubation. However, there was higher biomass production in quinoa than in lentils. Substrates containing filamentous fungal biomass can be considered added-value ingredients for food and feed recipes because this biomass is rich not only in high biological value proteins but also in polyunsaturated fatty acids, minerals, vitamins and pigments. In addition, scholars have noted the potential of using filamentous fungal biomass as a prebiotic because of the fungal cell wall polysaccharides (Karimi et al. 2021).

To evaluate the impact of biomass growth on the protein of the fermented samples, total protein content and the soluble protein fraction in TCA were evaluated at different times of the bioprocess (Table 2). Consistent with the biomass growth on the different substrates, a positive correlation between biomass and protein content was observed. Protein content increased by between 7 and 26% depending on the substrate. The highest production was found in quinoa seeds (26%), followed by lentil flour (21%). The impact of the particle size of the substrate on protein content is unclear and depends on the type of substrate because the increase of protein was higher in lentil flour than in grain, whereas, in quinoa, the opposite was observed. However, soluble protein decreased with fermentation time and was greater in quinoa seeds and flour than in lentils. An increase of total protein content has been reported in SSF with *P. ostreatus* in other pulses, such as kidney beans (13%), black beans (*Phaseolus vulgaris*; 6%; Espinosa-Páez et al. 2017) and *Lens culinaris* lentils (18.5%), a different lentil variety from the one used in this study. This protein increase could be explained by the fact that, during fermentation, carbohydrates serve as an energy source for fungus growth, and some of them may be bioconverted into complex proteins, peptides or even free amino acids (Asensio-Grau et al. 2020). Similar results were found by Mora-Uzeta et al. (2019), who observed that protein content increased in tepary beans (*Phaseolus acutifolius*) by more than 35% when fermented by *Rhizopus oligosporus*. Regarding the effect of fermentation on the soluble protein fraction, the scientific literature reports differing results depending on the substrate and/or inoculum employed. Asensio-Grau et al. (2020) reported an increase of this fraction after SSF with *P. ostreatus* in *Lens culinaris* lentils, contributing to higher digestibility of the resulting flour. In contrast, SSF with *Aspergillus sojae* and *ficuum* resulted in a proteolysis reduction in lupin flour due to the entrapment of smaller protein fractions in the fibrous matrix (Olukomaiya et al. 2020).

**Table 2:** Total and soluble protein contents in lentil grain, quinoa seeds and respective flours at different fermentation times

Fermentation time (days)	Total protein content (g protein/100 g dry basis) and soluble protein fraction in TCA (g soluble protein/100 g protein)			
	Lentil grain (LG)	Lentil flour (LF)	Quinoa seeds (QS)	Quinoa flour (QF)
0	24.4 ± 1.5^abc^ (12.7 ± 0.7^d^)	27.5 ± 0.9^a^ (12.75 ± 0.15^ g^)	20 ± 2^a^ (14.30 ± 1.15^c^)	25.5 ± 0.7^a^ (18.5 ± 0.5^c^)
2	24.5 ± 0.7^abc^ (12.1 ± 0.7^ cd^)	29.0 ± 0.3^b^ (11.0 ± 0.2^f^)	20.4 ± 1.0^ab^ (13.1 ± 0.4^b^)	27.7 ± 0.9^b^ (20.0 ± 1.3^d^)
4	25.5 ± 1.3^c^ (11.3 ± 0.3^abc^)	30.8 ± 0.4^c^ (10.50 ± 0.04^e^)	21.7 ± 0.4^bc^ (11.1 ± 0.4^a^)	28.2 ± 0.6^b^ (16.67 ± 0.15^b^)
6	25.1 ± 0.6^bc^ (11.3 ± 0.5^abc^)	30.3 ± 0.3^c^ (10.34 ± 0.12^e^)	22.5 ± 0.2^ cd^ (11.5 ± 0.2^a^)	30.8 ± 0.3^d^ (16.1 ± 0.3^b^)
8	24.7 ± 0.4^bc^ (10.6 ± 0.2^a^)	30.48 ± 0.15^c^ (10.04 ± 0.05^d^)	22.9 ± 0.3^ cd^ (12.6 ± 0.4^b^)	29.36 ± 0.10^c^ (14.9 ± 0.2^a^)
10	24.5 ± 0.6^abc^ (11.8 ± 0.4^bc^)	30.4 ± 0.7^c^ (8.86 ± 0.02^b^)	23.8 ± 0.8^de^ (11.1 ± 0.2^a^)	29.3 ± 0.7^c^ (14.6 ± 0.4^a^)
12	23.1 ± 1.0^a^ (11.1 ± 0.6^ab^)	31.9 ± 0.5^d^ (8.26 ± 0.14^a^)	24.4 ± 0.4^ef^ (11.5 ± 0.5^a^)	29.36 ± 0.09^c^ (14.8 ± 0.3^a^)
14	23.8 ± 0.5^ab^ (11.5 ± 0.5^bc^)	33.3 ± 0.2^e^ (9.37 ± 0.09^c^)	25.2 ± 0.5^f^ (11.0 ± 0.3^a^)	27.4 ± 0.7^b^ (14.7 ± 0.3^a^)

The results represent the mean of three repetitions with their standard deviation. ^abcdef^ lowercase letters indicate significant differences between the different fermentation times at the 95% (*p* < 0.05) significance level

^*^Values in parentheses correspond to soluble protein fraction in TCA

**Fig. 2:**
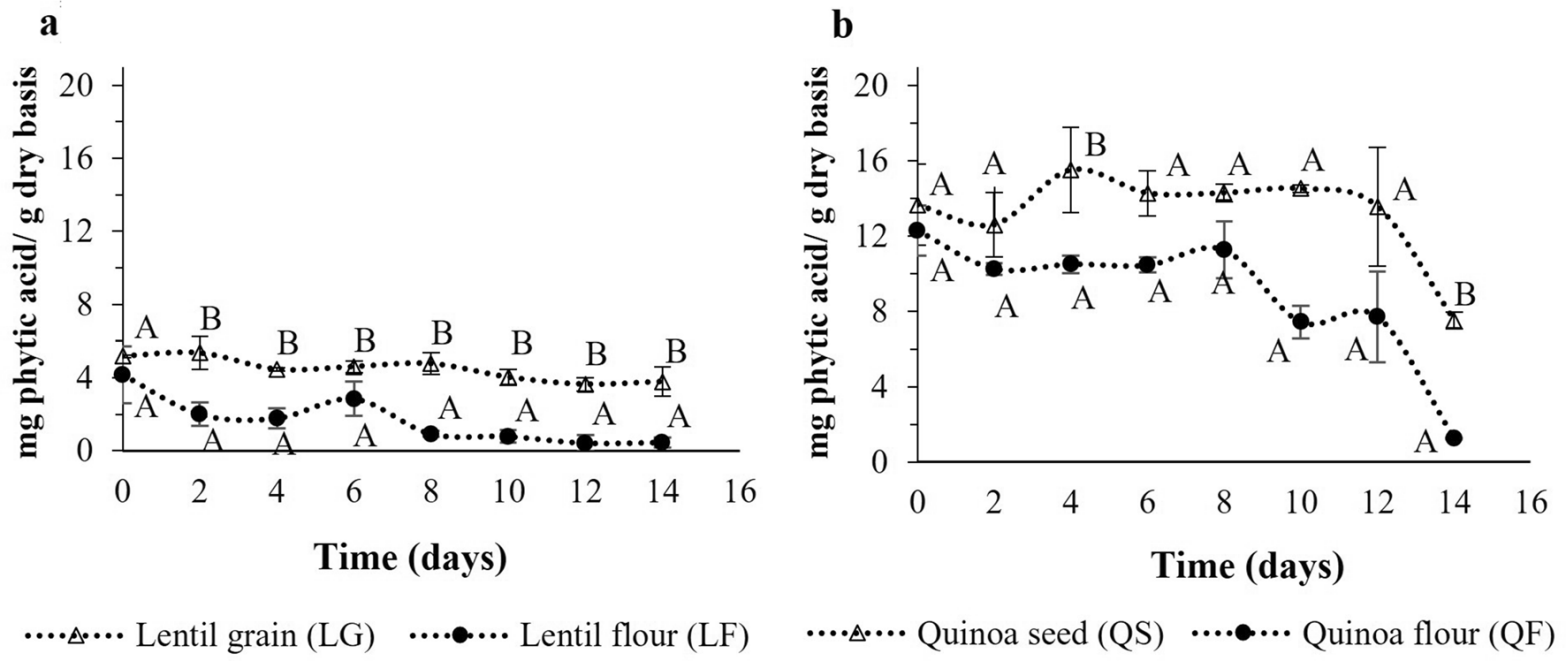
Phytic acid content in lentil grain, quinoa seeds and respective flours at different fermentation times. ^AB^ Capital letters indicate significant differences between grain/seeds and flour at the 95% (*p* < 0.05) significance level

An important aspect of the nutritional evaluation of a food or ingredient is the content of some antinutrient compounds. Phytates are known to contribute to decreasing the absorption of essential micronutrients, such as calcium, iron (Hurrell et al. 2003), zinc (Guttieri et al. 2006) and magnesium (Bohn et al. 2004; Peng et al. 2010). They also have a negative impact on protein digestibility because they can bond to dietary protein or digestive enzymes (proteases and amylases), inhibiting their hydrolytic activity (Espinosa-Páez et al. 2017; Muñoz-Llandes et al. 2019). Because fungal SSF is presented as a strategy to reduce the antinutrient content of certain substrates (Garrido-Galand et al. 2021), the evolution of phytic acid content was monitored during the fermentation process. The results are shown in Fig. 2. According to the literature, quinoa seeds contain approximately 1% to 2% of phytic acid (Hídvégi and Lásztity 2002; Febles et al. 2002), whereas lentils contain between 0.3 and 1.5%. These values are consistent with the initial values for the raw materials used in this study (Table 1).

The degradation of phytates as a consequence of fungal fermentation was observed. This degradation was more pronounced in flours than in grains. The percentage reduction was 27% and 89% in lentil grain and flour, respectively. In quinoa, the percentage reduction was 45% in seeds and 90% in flour. These changes began to be significant (*p* < 0.05) from the 10th day of fermentation and depended on the substrate characteristics. The degradation of phytates after fermentation was greater in flours than in grains. These results are in accordance with those of Castro-Alba et al. (2019), who reported different levels of phytate degradation in quinoa, canihua and amaranth according to their granulometry (seeds or flour). The degradation rate of phytates also seems to be moderated by the pH reduction during fermentation because of organic acid production, which depends on the inoculum employed. Castro-Alba et al. (2019) reported differences between spontaneous fermentation and fermentation with characterised species, such as *L. plantarum*. Furthermore, they suggested that the greater degradation of phytates in flour depends on both the exogenous phytase production of the microorganism and the activation of endogenous phytase of the substrate. However, autoclaving prior to inoculation causes the inactivation of endogenous phytase (Brejnholt et al. 2011). Therefore, the notable decrease in phytates in the last few days of fermentation may be attributed mainly to the activity of the exogenous phytase from *P. ostreatus*, instead of the action of the endogenous phytase of the substrate. Similar results were found by Liang et al. (2008), who reported that fermentation of brown rice was more effective in decreasing phytic acid than wet heating at 115 °C for 10 min.

**Fig. 3:**
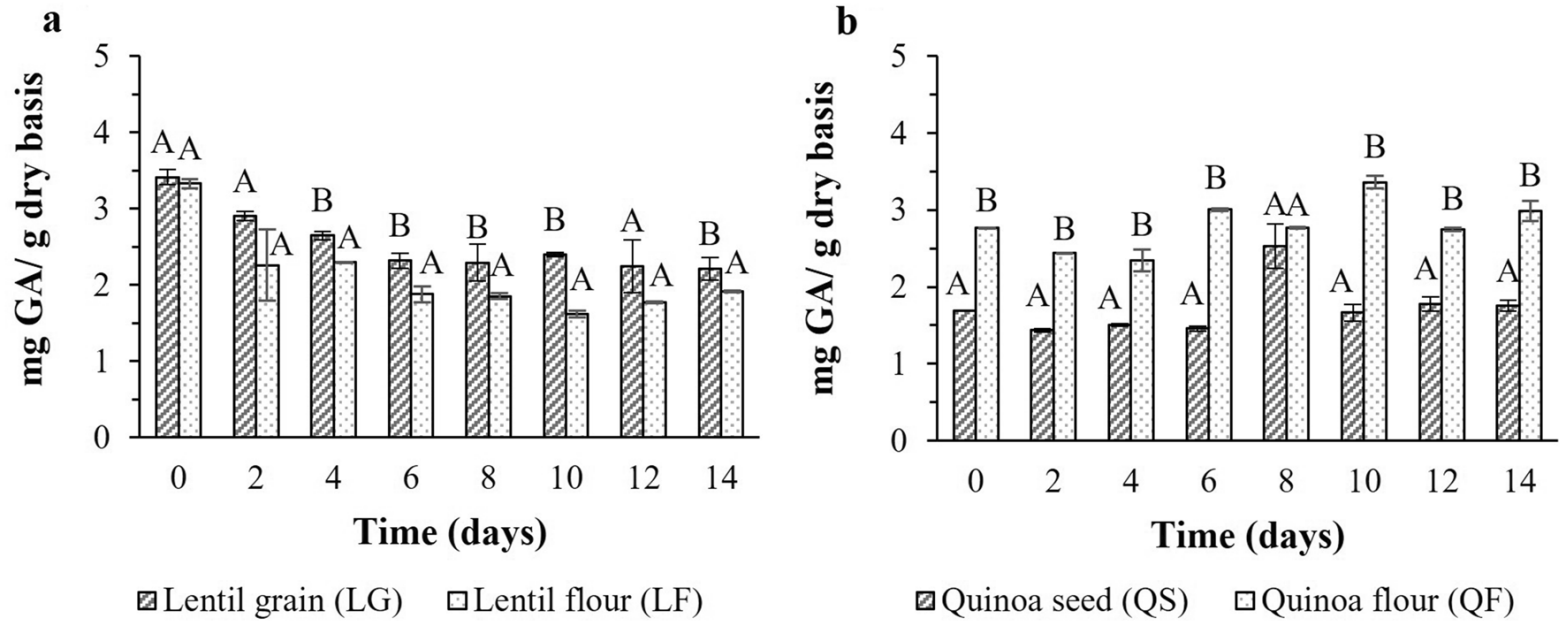
Total phenol content in lentil grain and quinoa seeds and respective flours at different fermentation times. ^AB^ Capital letters indicate significant differences between grain/seeds and flour at the 95% (*p* < 0.05) significance level

*Pleurotus ostreatus* is also known to be an excellent producer of hydrolytic enzymes, which contribute to the release of conjugated phenolic compounds chelated into the cell walls by hydrolysis during fermentation. Phenolic compounds are the major contributors to antioxidant activity in fruit, vegetable, grain and plant tissues. Changes in total phenol content (TPC) with SSF time are shown in Fig. 3. First, the results show a positive impact of thermal treatment on bound phenolic compound release because TPC was much higher after autoclaving (time 0) than in the raw material (Table 1). The data agree with those reported by other authors (Bryngelsson et al. 2002; Madapathage Dona 2011). Hence, thermal treatment could promote cell wall disruption with the release of structural phenols and/or the breakdown of insoluble polymeric phenols into smaller molecular weight compounds with enhanced extractability.

In contrast, a decreasing profile of TPC was observed as the fermentation progressed in lentil substrates, with a higher TPC in flour than in grain. Gebru and Sbhatu (2020) reported similar findings in white and brown teff subjected to SSF with *P. ostreatus*, with a negligible and even slight decrease of TPC after 6 days. However, same authors reported a significant increase of teff phenols when *G. lucidum* was used as a starter and under the same SSF conditions. This result highlights the relevance of each fungal mycelium metabolism and enzyme synthesis in producing changes in bioactive compounds.

Along these lines, a negative correlation between TPC and fermentation time was observed by Xu et al. (2018) in eight cereals and pseudocereals (wheat, corn, rice, millet, quinoa, oats, sorghum and buckwheat) and two legumes (soybean and peas) fermented with three different fungi for 35 days. According to their results, an increase of TPC was only observed at 14 days in oats. For the other substrates, longer fermentation times were required to produce a significant increase of TPC content. For instance, Xu et al. (2018) reported a significant increase of TPC in fermented quinoa from 21 days of fermentation. This finding may explain the low TPC increase observed in the present study. Enzyme production is known to change over time, affecting the transformation and production of particular compounds. Therefore, optimal enzyme production is obtained at a specific time in a given culture. The highest TPC value in quinoa seeds was detected after eight days of fermentation (2.5 ± 0.3 mg GA/g dry basis), whereas, in flour, the highest value was observed after 10 days (3.36 ± 0.08 mg GA/g dry basis).

The antioxidant activities of fermented lentils and quinoa appear in Table 3. Although SSF was indicated with the aim of obtaining new ingredients with enhanced antioxidant properties, the capacity of the fermented substrates to scavenge free radicals, such as ABTS and DPPH, and to reduce ferric ions in the FRAP assay decreased with fermentation time in this study. Greater losses of antioxidant activities were observed in lentils than in quinoa. In addition to total phenols, other metabolites, such as ergothioneine, that formed during the fermentation process may affect the antioxidant properties of the fermented products (Cai et al. 2012; Zhai et al. 2015; Bei et al. 2017). This fact may be responsible for the lack of correlation between TPC and antioxidant activity (Torino et al. 2013; Magro et al. 2019). In addition, competitive reactions between prooxidant and antioxidant compounds can occur, resulting in an increase or reduction of a food’s antioxidant capacity. The ability of phenolic compounds to promote or inhibit oxidative damage depends on the phenol concentration and pH, amongst other factors. Monohydroxylated phenols have been reported to exhibit low radical scavenging activity (Briante et al. 2003; Villaño et al. 2005). Fukumoto and Mazza (2000) found that benzoic and cinnamic acid derivatives behave like prooxidants. Accordingly, a higher release of prooxidant phenols occurred in lentils than in quinoa as fermentation time increased.

**Table 3:** Antioxidant activity in lentil grain, quinoa seeds and respective flours at different fermentation times

Substrate	Fermentation time (days)	Antioxidant activity (mg Trolox/g dry basis)		
		ABTS	DPPH	FRAP
Lentil grain (LG)	0	10.7 ± 0.5^dA^ (0 ± 5)	5.6 ± 0.5^fB^ (0 ± 9)	7.3 ± 0.3^fA^ (0 ± 5)
	2	9.7 ± 0.4^bcA^ (− 9 ± 4)	4.4 ± 0.2^eB^ (− 21 ± 3)	4.8648 ± 0.0012^eA^ (− 33.30 ± 0.02)
	4	8.7 ± 0.2^aA^ (− 19 ± 2)	3.92 ± 0.08^dB^ (− 30.1 ± 1.5)	4.17 ± 0.06^dB^ (− 42.9 ± 0.9)
	6	9.3 ± 0.2^abA^ (− 13 ± 2)	3.1 ± 0.2^cB^ (− 44 ± 3)	3.03 ± 0.11^cB^ (− 58.5 ± 1.6)
	8	8.99 ± 0.12^aA^ (− 16.3 ± 1.2)	2.5 ± 0.2^abB^ (− 55 ± 3)	2.31 ± 0.08^bB^ (− 68.3 ± 1.1)
	10	10.2 ± 0.2^cdB^ (− 5 ± 2)	2.79 ± 0.12^bcB^ (− 50 ± 2)	2.54 ± 0.03^bB^ (− 65.2 ± 0.5)
	12	9.9 ± 0.9^bcA^ (− 7 ± 8)	2.21 ± 0.11^aB^ (− 61 ± 2)	1.8 ± 0.2^aB^ (− 75 ± 3)
	14	10.0 ± 0.3^bcA^ (− 7 ± 3)	2.15 ± 0.03^aB^ (− 61.7 ± 0.6)	1.72 ± 0.14^aB^ (− 76 ± 2)
Lentil flour (LF)	0	12.90 ± 0.02^dB^ (0.00 ± 0.13)	4.307 ± 0.006^fA^ (0.00 ± 0.14)	7.53 ± 0.06^fA^ (0.0 ± 0.8)
	2	11.1 ± 0.8^cA^ (− 14 ± 6)	3.1 ± 0.3^eA^ (− 29 ± 7)	4.5 ± 0.6^eA^ (− 41 ± 8)
	4	10.90 ± 0.12^cB^ (− 15.5 ± 0.9)	2.65 ± 0.08^dA^ (− 39 ± 2)	3.68 ± 0.11^dA^ (− 51 ± 2)
	6	10.3 ± 0.2^abB^ (− 20.1 ± 1.5)	2.06 ± 0.02^cA^ (− 52.2 ± 0.5)	2.52 ± 0.15^cA^ (− 67 ± 2)
	8	10.06 ± 0.02^aB^ (− 22.1 ± 0.2)	1.872 ± 0.014^bA^ (− 56.5 ± 0.3)	1.92 ± 0.06^bA^ (− 74.5 ± 0.7)
	10	9.81 ± 0.08^aA^ (− 24.0 ± 0.6)	1.54 ± 0.02^aA^ (− 64.3 ± 0.5)	1.19 ± 0.04^aA^ (− 84.2 ± 0.6)
	12	9.99 ± 0.10^aA^ (− 22.6 ± 0.8)	1.6087 ± 0.0008^aA^ (− 62.65 ± 0.02)	1.43 ± 0.03^aA^ (− 81.0 ± 0.5)
	14	10.67 ± 0.13^bcB^ (− 17.3 ± 1.0)	1.66 ± 0.05^aA^ (− 61.5 ± 1.1)	1.36 ± 0.12^aA^ (− 82 ± 2)
Quinoa seeds (QS)	0	11.54 ± 0.04^bB^ (0.0 ± 0.3)	3.08 ± 0.02^eB^ (0.0 ± 0.7)	2.74 ± 0.15^dA^ (0 ± 5)
	2	10.84 ± 0.04^aB^ (− 6.1 ± 0.3)	2.76 ± 0.09^dB^ (− 10 ± 3)	1.75 ± 0.03^bA^ (− 36.2 ± 1.2)
	4	10.84 ± 0.04^aB^ (− 6.1 ± 0.3)	2.576 ± 0.015^cB^ (− 16.3 ± 0.5)	1.98 ± 0.05^cA^ (− 28 ± 2)
	6	10.84 ± 0.03^aB^ (− 6.1 ± 0.3)	2.48 ± 0.04^bcB^ (− 19.3 ± 1.4)	1.55 ± 0.03^aA^ (− 43.6 ± 1.0)
	8	12.83 ± 0.07^cB^ (11.1 ± 0.6)	2.30 ± 0.11^aA^ (− 25 ± 4)	1.44 ± 0.07^aA^ (− 47 ± 3)
	10	11.40 ± 0.08^bB^ (− 1.3 ± 0.7)	2.28 ± 0.10^aA^ (− 26 ± 3)	1.5 ± 0.2^aA^ (− 45 ± 8)
	12	11.7 ± 0.4^bB^ (2 ± 3)	2.45 ± 0.02^bB^ (− 20.3 ± 0.7)	1.42 ± 0.08^aA^ (− 48 ± 3)
	14	10.7 ± 0.4^aB^ (− 7 ± 4)	2.555 ± 0.002^bcB^ (− 17.00 ± 0.06)	1.458 ± 0.004^aA^ (− 46.7 ± 0.2)
Quinoa flour (QF)	0	9.37 ± 0.03^cA^ (− 0.0 ± 0.4)	2.81 ± 0.04^eA^ (0.0 ± 1.4)	4.87 ± 0.06^fB^ (0.0 ± 1.3)
	2	8.908 ± 0.007^bA^ (− 4.94 ± 0.07)	2.55 ± 0.03^dA^ (− 9.2 ± 1.2)	3.75 ± 0.08^eB^ (− 23 ± 2)
	4	8.5 ± 0.2^aA^ (− 10 ± 2)	1.98 ± 0.06^aA^ (− 30 ± 2)	2.4 ± 0.3^cB^ (− 50 ± 5)
	6	9.36 ± 0.04^cA^ (− 0.2 ± 0.5)	2.291 ± 0.008^cA^ (− 18.4 ± 0.3)	2.77 ± 0.02^dB^ (− 43.0 ± 0.3)
	8	9.60 ± 0.13^deA^ (2.4 ± 1.4)	2.39 ± 0.02^cA^ (− 15.0 ± 0.8)	2.31 ± 0.08^bcB^ (− 53 ± 2)
	10	9.44 ± 0.09^cdA^ (0.7 ± 1.0)	2.12 ± 0.05^bA^ (− 24 ± 2)	1.97 ± 0.11^aB^ (− 60 ± 2)
	12	9.516 ± 0.002^cdeA^ (1.55 ± 0.02)	2.09 ± 0.14^bA^ (− 26 ± 5)	2.34 ± 0.02^bcB^ (− 51.8 ± 0.4)
	14	9.7 ± 0.2^eA^ (3 ± 3)	2.16 ± 0.06^bA^ (− 23 ± 2)	2.21 ± 0.05^bB^ (− 54.5 ± 1.0)

Values in parentheses correspond to the percentage of variation with respect to non-inoculated substrate (time 0). The results represent the mean of three repetitions with their standard deviation. ^abcde^Lowercase letters indicate significant differences between the different fermentation times at the 95% (*p* < 0.05) significance level. ^AB^ Capital letters indicate significant differences between grain and flour at the 95% (*p* < 0.05) significance level

Nevertheless, autoclaving was once again observed to have a positive effect in terms of radical scavenging and reducing power activity of both substrates because a notable increase in milligrams of Trolox per gram of dry basis at time 0 was observed compared to the values found in the raw materials (Table 1). These findings are in line with those of Rocchetti et al. (2019).

Despite the results, complementary analysis is needed to determine how down-streams unit operations, such as milling and drying, usually applied by the food industry to obtain stable flours can affect the studied parameters. Also, it would be of interest to analyse changes in the studied parameters with in vitro gastrointestinal digestion to establish the added value of SSF in terms of not only compositional variation but also protein and carbohydrate digestibility and bioactive compound bioaccessibility.

## Conclusions

Solid-state fermentation (SSF) with *P. ostreatus* has been proved to be an efficient way to enhance the nutritional profile of Pardina lentils and white quinoa in terms of increased protein and reduced phytates contents. Those nutritional changes along with the additional potential health benefits due to the presence of fungal biomass support the bioconversion of legumes and pseudocereals by SSF. Therefore, this bioprocess may be considered an environmentally sustainable biotechnological strategy to obtain gluten-free fermented lentil- and quinoa-based ingredients for novel food formulations that target specific population groups with high protein requirements, such as the elderly, athletes, vegans or individuals with gastrointestinal disorders. It would be of interest to perform in vitro digestion studies that could help with decisions to establish optimal conditions for the production of fermented ingredients with enhanced digestibility. In conclusion, this study contributes to different twenty-first century food technology challenges related to protein diversification and the environmentally sustainable bioproduction of food ingredients.

## Data Availability

All data generated or analysed during this study are included in this article (and its additional information files).

## Abbreviations

ABTS: 2,2′-Azino-bis (3-ethylbenzothiazoline-6-sulphonic acid)
CECT: Spanish type culture collection
°C: Degrees celsius
DPPH: 2,2-Diphenyl-1-picrylhydrazyl
FRAP: Ferric reducing antioxidant power
g: Gram
GA: Gallic acid
l: Litre
LF: Lentil flour
LG: Lentil grain
mg: Milligram
QF: Quinoa flour
QS: Quinoa seed
rpm: Revolutions per minute
SmF: Submerged fermentation
SSF: Solid-state fermentation
TCA: Trichloroacetic acid
TPTZ: 2,4,6-Tripyridyl-s-triazine complex
UV: Ultraviolet
V: Volume
Vis: Visible
W: Weight
